# Feasibility of a mental practice intervention in stroke patients in nursing homes; a process evaluation

**DOI:** 10.1186/1471-2377-10-74

**Published:** 2010-08-24

**Authors:** Susy M Braun, Jolanda C van Haastregt, Anna J Beurskens, Alexandra I Gielen, Derick T Wade, Jos M Schols

**Affiliations:** 1 Research Centre for Autonomy and Participation of Persons with a Chronic Disease, Department of Health and Technique, Zuyd University of Applied Sciences, Heerlen, The Netherlands; 2 Centre of Expertise in Life Sciences, Zuyd University of Applied Sciences, Heerlen, The Netherlands; 3 CAPHRI School for Public Health and Primary Care, Maastricht University, Maastricht, The Netherlands; 4 Department of Health Care and Nursing Science, Faculty of Health, Medicine and Life Sciences, Maastricht University, Maastricht, The Netherlands; 5 Klevarie Nursing Home, Vivre foundation, Maastricht, The Netherlands; 6 Department of Health and Care, Zuyd University of Applied Sciences, Heerlen, The Netherlands; 7 Department of Rehabilitation, Faculty of Health, Medicine and Life Sciences, Maastricht University, Maastricht, The Netherlands; 8 Oxford Centre for Enablement, Oxford, Great-Britain; 9 Department of General Practice, Faculty of Health, Medicine and Life Sciences Maastricht University, Maastricht, The Netherlands

## Abstract

**Background:**

Within a multi-centre randomised controlled trial in three nursing homes, a process evaluation of a mental practice intervention was conducted. The main aims were to determine if the intervention was performed according to the framework and to describe the therapists' and participants' experiences with and opinions on the intervention.

**Methods:**

The six week mental practice intervention was given by physiotherapists and occupational therapists in the rehabilitation teams and consisted of four phases: explanation of imagery, teaching patients how to use imagery, using imagery as part of therapy, and facilitating the patient in using it alone and for new tasks. It had a mandatory and an optional part. Data were collected by means of registration forms, pre structured patient files, patient logs and self-administered questionnaires.

**Results:**

A total of 14 therapists and 18 patients with stroke in the sub acute phase of recovery were involved. Response rates differed per assessment (range 57-93%). Two patients dropped out of the study (total n = 16). The mandatory part of the intervention was given to 11 of 16 patients: 13 received the prescribed amount of mental practice and 12 practiced unguided outside of therapy. The facilitating techniques of the optional part of the framework were partly used. Therapists were moderately positive about the use of imagery in this specific sample. Although it was more difficult for some patients to generate images than others, all patients were positive about the intervention and reported perceived short term benefits from mental practice.

**Conclusions:**

The intervention was less feasible than we hoped. Implementing a complex therapy delivered by existing multi-professional teams to a vulnerable population with a complex pathology poses many challenges.

## Background

Stroke is one of the leading causes of death and acquired disabilities globally [1]. Treating patients after stroke is challenging: patients are often very vulnerable, especially in the sub acute phase of recovery, and stroke is a complex pathology that can lead to a variety of symptoms. Much research on effectiveness of treatments within stroke has been performed, but there is no evidence supporting most specific rehabilitation treatments [2]. Only a small percentage of the day in organized stroke units is spent with therapists [3], which may not be optimal as a higher amount of practice is known to be related to more improvement [4,5].

Mental practice of tasks is a relatively new therapy that is receiving increasing attention within rehabilitation research. Practicing a skill mentally is potentially a method to increase the amount of therapy during rehabilitation in a safe way with relatively low costs. Mental practice has been defined in many ways, and our definition [6] used for this study was *a training or therapy form in which an internal representation of the movement is activated and the execution of the movement repeatedly mentally simulated, without physical activity, within a chosen context*. There is some evidence that mental practice might be effective in patients with stroke [6-8].

In the Netherlands, selected younger and less cognitively impaired minority of patients are admitted to stroke rehabilitation centres, but the majority of stroke healthcare takes place in nursing homes in which patients are treated by multidisciplinary teams according to stroke guidelines [1,9,10]. In a randomized controlled trial, we assessed the effects of mental practice on recovery of stroke patients in three Dutch nursing homes. The mental practice framework developed [11] consisted of a mandatory and an optional part. The intervention was given by physiotherapists and occupational therapists.

As part of this randomized controlled trial we performed a process evaluation. The aims of this study were to determine if the mental practice intervention was delivered according to the framework, and to evaluate the therapists' and participants' experiences with and opinions on the intervention.

## Methods

### Study design and population

This process evaluation is a descriptive study in which both quantitative and qualitative data were gathered. The study was approved by the local medical ethical committee (METC Atrium MC, HsZuyd, Orbis Medical Concern, internal number 07-T-18). The study sample consisted of all individuals with stroke from three Dutch nursing homes allocated to the experimental, mental practice group of the trial and all the therapists who performed the intervention. Details of the design of the randomized controlled trial are presented elsewhere ([12], http://www.trialregister.nl | NTR993).

### The intervention studied

We developed a mental practice framework which allowed therapists and patients to tailor the intervention to the patients needs [11]. A flexible protocol was used because research tells us that rehabilitation following stroke should be tailored to the preferences and abilities of each individual seen, especially when patients have complex problems such as seen in people after stroke. This study was set in the context of regular clinical (non-research) rehabilitation practice, and so the framework was developed to be embedded in regular therapy sessions of 30 minutes where the actual allocation of time to specific aspects of therapy necessarily varied according to the patient's needs and wishes.

The framework has been described in detail [11] and it has four phases: explanation of imagery, teaching patients how to use imagery, using imagery as part of therapy, and facilitating the patient in using it alone and for new tasks.

#### Explanation

Mental practice was explained to the patient by the therapist; what it is, how it might help and what it cannot do (to avoid unrealistic expectations). In addition, skills which the patients wanted to improve were chosen with the patient. Apart from 'drinking' and 'walking', which were the same for all patients, patients could choose two other skills with their occupational therapist and physiotherapist: one for the arms and one for the legs.

#### Teaching

In this phase patients were first taught to imagine the sequences of movement needed to achieve the task set, with an emphasis upon achieving the correct sequence. Then they were taught how to maximize the vividness (reality) of the imagined experience. Teaching involved alternation of physical and mental practice, using the physical practice to generate a correctly sequenced, vivid mental representation. The balance varied according to a patient's needs. Each patient could choose the representation (first or third person) that they found easiest [13-15]. In the original description [11], nine optional facilitating techniques were given to be used to assist this training.

The success of a patient in imagining the actions correctly and vividly was judged by self-report, checked as far as possible by comparing the time taken to perform a task mentally against the time in reality, and by checking that the patient could recite the order of actions correctly.

#### Use in practice

Once the therapist judged that the patient had learned how to practice mentally, it was incorporated into therapy. Patients were asked to use it primarily before attempting the task concerned (outside therapy sessions as well). Furthermore as patients reported that imagining the separate actions was becoming easier, they were encouraged to practice groups of actions and eventually the whole task as a single unit, so-called chunking [16].

#### Self-directed use

As patients gained confidence, needing less support and reassurance, the therapist reduced the frequency and extent of the support offered. They also encouraged the patient to develop its use for other tasks.

Both physiotherapists and occupational therapists worked according to the framework, but often they were working on different tasks such as using stairs and feeding with a spoon. The mental practice intervention was given for six weeks and consisted of a mandatory and an optional part. The mandatory part of the framework was determined by defining the minimum a patient should receive mental practice to have a plausible effect. This amount was based on averages in literature in which effect of mental practice interventions were still found [6].

### The mandatory part

•After mental practice was explained (phase 1), the patients were required to receive at least ten mental practice training sessions embedded in regular therapy spread over at least three weeks. These sessions were to be guided by a trained therapist.

•As soon as the therapists thought patients were able to perform imagery outside of therapy they were required to encourage unguided mental practice at once. When the therapist thought patients were able to practice correctly outside of therapy, logs (one per week) had to be handed to the patients to record unguided mental practice behaviour. In principle, a maximum of six logs could be filled in.

### The optional part

In the framework [11] nine facilitating techniques to teach imagery were given: talking through or verbalising the movement by either (1) the patient or (2) the therapist; (3) enhancing sensory information by passively moving a body limb or joint; (4) giving visual cues by watching clips of movements from a DVD; (5) observing oneself in the mirror; (6) observing others (including demonstration by the therapist); (7) using audio cues; (8) using visual cues; and, (9) extracting a part of a movement from the entire skill to practice mentally (e.g. placing the foot within a step) before embedding the part back into the entire skill.

### Training of therapists

In each nursing home, therapists were trained in teaching patients about mental practice through a presentation of the theoretical background at two half day workshops, practical work with patients, and group discussions. The therapists received written instruction (course map, selected background literature) and additional materials to facilitate performing mental practice (e.g. patient logs, pre structured patients files, video clips of daily movements). Therapists could always call for additional help of the researcher (SB) during the trial.

### Data collection

Data from the therapists were collected by means of registration forms and pre structured patient files in which therapists recorded the frequency and content of therapy and also the clinical progress of the patient (mandatory part guided imagery). Patients were asked to keep a log of the use of mental practice outside of therapy (mandatory part unguided imagery), and to record their mood and anything else of note during the intervention period.

At the end of the trial the therapists filled out a questionnaire (therapists' experiences and opinion). Patients were handed a self-administered questionnaire after their six weeks of treatment (patients' experiences and opinion).

### Data analysis

Data from the pre structured patients' files and logs were extracted by a researcher (SB). Quantitative data from the questionnaires were analysed and put into frequency tables and open comments were clustered in themes. Quotes were used to illustrate the main results.

## Results

The study patient sample consisted of five male and 13 female patients with an average age of 77.7 years (SD 7.2 years). Patients were included between the second and 10^th ^week post-stroke with the average being six weeks (SD 2.7 weeks) post stroke at the beginning of the intervention period. During the intervention period two of the 18 patients dropped out of the randomized study: one of these two perceived mental practice as not worthwhile, the other left for reasons unrelated to the intervention.

The study therapist sample consisted of all therapists giving the intervention: eight physiotherapists and six occupational therapists. They were already specialised in stroke rehabilitation and most had some awareness of mental practice but did not use it regularly. Of the 14 therapists taught, one physiotherapist did not participate in the entire trial because he started working elsewhere and one occupational therapist missed a part because of maternity leave.

Data collection was reasonably complete. In one nursing home the data on the amount of mental practice treatment was not available because therapists partly used their own patient files; this affected the registered amount of mental practice treatment per step of the framework in two patients. The response rates of the different measurement instruments are presented in table 1.

**Table 1:** Overview of data collection group sizes and response rates for the different measurement instruments

Measurement instrument	Use of measurement instrument (aim)	Attending, completed or received	Attending rate, Response rate	Comments
**Pre structured patient files **Number of patients treated: 16	Determine delivery of mandatory part of framework (step reached and amount of therapy sessions)	Patient files (n = 14)	Response rate: 88%	*Two patients dropped out before ending of six week intervention, leaving 16 patients treated. In another two patients the pre structured registration form was not used.14 patient files were therefore checked*.
**Patient logs**	Determine delivery of mandatory part of framework (use of unguided MP outside of therapy)	Filled in correctly (n = 58) by 12 participants	Response rate: 75%	*Twelve out of 16 patients used a log for one or more weeks, at average five logs per patient during 6 weeks*.
**Questionnaire therapists at end of the trial** Number of questionnaires handed out: 14	Assess therapists' opinions and experiences with MP	Completed (n = 7), but 11 therapists contributed	Response rates: Therapists: 79%, Questionnaires: 57%	*One questionnaire was filled in by 2 therapists, and another by four therapists*
**Questionnaire patients after six week intervention **Number of questionnaires handed out: 15	Assess patients' opinions and experiences with MP	Completed (n = 14)	Response rate: 93%	*One patient did not receive a questionnaire (forgotten). Of the 15 questionnaires given out, one was not completed*.

MP = mental practice

### Therapy amount; showing up for therapy

The 16 patients generally attended their allocated therapy sessions. In three pre structured patient files a short illness period due to influenza was reported, interrupting the intervention for two days in phase two (n = 2) or phase 3 (n = 1). Occasionally a therapy session was cancelled because of a doctor's appointment at the hospital, but the amount of therapy 'lost' was small. The number of treatments given to an individual patient ranged from 10 to 48 therapy sessions, with an average of 24 (SD 9.8) sessions in six weeks.

### Performance according to framework; the mandatory part within therapy time

Table 2 shows that thirteen (93%) out of the 14 patients with full information received the obligatory amount of at least 10 sessions of mental practice (phase 2): three participants completed the entire framework, eight were in step three after six weeks and step one and two were achieved in three patients. One patient did not receive the required amount of practice therapy as determined in the mandatory part. It is notable that the last step of the framework was rarely reached.

**Table 2:** Overview of the mandatory part of the mental practice framework; guided (left) and unguided imagery (right).

Home A, B, C and patient number	Data on guided imagery (total within therapy during six weeks intervention period)						Data on unguided imagery (total outside of therapy time over six weeks intervention period)					Both mandatory parts received (yes/no)
		
	Total number of treatments	Treatments Step 1	Treatments step 2	Treatments step 3	Treatments step 4	Mandatory part of guided MP (yes/no)	Total number of logs (max. 6)	Amount days practiced (max. 42)	Number MP sessions	Total amount minutes practiced	Mandatory part of unguided MP (yes/no)	
A -101	24	4	15	4	1	Yes	6	35	95	683	Yes	Yes
A -105	35	2	18	15	0	Yes	1	4	10	100	Yes	Yes
A -106	19	2	13	4	0	Yes	6	23	23	940	Yes	Yes
A -109	33	4	29	0	0	Yes	6	33	136	1751	Yes	Yes
A -110	27	4	23	0	0	Yes	0	0	0	0	No	No
A -113	10	4	6	0	0	No	6	37	49	284	Yes	No
A -114	48	6	12	30	0	Yes	5	25	47	530	Yes	Yes
A -118	30	2	13	15	0	Yes	4	18	47	820	Yes	Yes
B -201	35	3	13	18	1	Yes	6	41	121	1098	Yes	Yes
B -206	18	6	6	6	0	Yes	0	0	0	0	No	No
B -207	19	6	2	11	0	Yes	6	37	94	1060	Yes	Yes
B -210	17	3	9	4	1	Yes	3	10	14	120	Yes	Yes
B -211	16	5	3	8	0	Yes	5	17	20	162	Yes	Yes
C -302	15	?	?	?	?	?	0	0	0	0	No	No
C -304	15	?	?	?	?	?	0	0	0	0	No	No
C -306	30	4	4	22	0	Yes	4	5	7	70	Yes	Yes

Total	386	55	166	137	3	14	58	285	663	7618	16	16

Average	24 SD 9.8					13 out of 14 (93%)	5	24	55 SD 45	635 SD 522	12 out of 16 (75%)	11 out of 16 (69%)

Left: the number of treatments per patient and per step in the protocol. Right: the number of logs used by the individual patients and number of days practiced outside of therapy during the six weeks of the intervention. The amount of sessions of unguided therapy and the amount of time spend on mental practice outside of therapy was recorded in minutes

? = no data available

The main reasons given for not completing all four steps of the framework were that therapists thought that patients did not understand what they were supposed to do, were unwilling to perform imagery or got frustrated by 'thinking too much'.

### Performance according to the framework; the mandatory part outside of therapy time

Twelve out of the sixteen patients (75%) practiced unguided outside of therapy within the six week intervention period (table 2). The remaining four participants did not return any of the logs or did not record practicing mentally outside of guided therapy. Among these four participants were also the two patients of whom no data on the number of treatments were recorded.

The remaining 12 participants filled in 58 logs (representing mental practice over five weeks), with a range from one to six logs completed. These 12 participants practiced a mean of 635 minutes (SD 522 minutes) during a mean of 55 unguided sessions (SD 45 sessions). Unguided mental practice sessions were recorded as being between two and 30 minutes long. The total minimum time over six weeks spent on mental practice outside of therapy was one hour and 10 minutes and the maximum was 29 hours and 11 minutes.

### The optional part of the framework

The frequency with which optional facilitating techniques to teach imagery techniques were used is shown in table 3. Only two were used frequently: 'talking through or verbalising the movement by the patient' and 'talking through and verbalising movement by therapist'.

**Table 3:** The use of the optional part of the mental practice framework; Overview of the most frequently used facilitating techniques by the therapists in the trial to teach and monitor imagery use

Home (A, B, C) and patient number	Facilitating techniques used (in %) during therapy sessions with therapists					
	VP	VT	VIC demo	VEC	EXT	VICM
A -101	67	21	8	0	0	0
A -105	0	14	6	6	0	0
A -106	42	16	0	0	0	0
A -109	67	15	0	0	0	0
A -110	11	15	0	0	0	0
A -113	90	80	0	0	0	0
A -114	48	42	0	8	0	0
A -118	40	40	0	30	0	0
B -201	51	3	0	0	0	0
B -206	28	0	0	0	39	0
B -207	32	0	0	0	0	0
B -210	47	0	0	0	0	0
B -211	0	63	0	0	0	0
C -302	?	?	?	?	?	?
C -304	?	?	?	?	?	?
C -306	33	100	0	0	0	17
Total	556	409	14	44	39	17
Average	51%	38%	1%	4%	4%	2%

VP = verbalising movement patient | VT = verbalising movement therapist | VICdemo = visual internal cues through demonstration | VEC = visual external cues | EXT = extracting parts of movement | VICM = visual internal cues through mirror observation | ? = no data recorded | A-nnn = nursing home (A, B or C) and patient number

### Therapists' experiences with the mental practice framework

The 12 therapists involved throughout all reported that the intervention was clear and if any problems occurred they contacted the primary researcher (SB) for assistance, which happened four times during the trial period. However, they admitted to gaining confidence with experience: '*When you however work longer with it or when you have had more patients, the framework is clear to work with*.' Ten therapists (86%) considered themselves capable of giving the mental practice intervention ('sufficient', n = 8; 'good', n = 2); two did not answer this question. Three therapists from the same location reported that they implemented mental practice within therapy time as planned. Seven therapists used between 10 and 20 additional minutes per therapy session and another two therapists were unable to estimate how much longer they used.

In the written comments, therapists reported that it was hard to check compliance of the patient. This may have lead to longer instruction times and seemed often to lead to a feeling of irritation (for the therapist). '*... but you just cannot check if they have practiced, which is very annoying*'. Another therapist was more optimistic: '*I believe that you do know who has practiced (the 'good' ones) and who has not (the 'bad' ones). However, there are a lot of patients that remain in a grey area in which you just don't know what they have done*'.

Therapists were also not sure whether the patients correctly registered the unguided mental training in the logs: '*Sometimes they would report what they had practiced physically outside of therapy or during therapy*.' Nonetheless all therapists thought that the patient logs were of some use in recording unguided imagery despite their doubts on the validity of the reported information.

### Therapists' opinion on benefits and use after the trial

Table 4 shows the results of the questionnaire to therapists on potential benefits, and their opinion of the role of mental practice in the future. Seven therapists reported they would use mental practice after the trial. However, four therapists doubted if they would use the entire framework or if they were going to use mental practice at all.

**Table 4:** Possible benefits stroke patients might gain from an additional mental practice intervention: opinions of eleven therapists

benefits patients might perceive through the use of MP as suggested by therapists	Yes, most patients	Yes, some patients	No, none of the patients
To Improve movements	2	9	-
To feel less anxious	1	5	5
To increase a secure feeling	1	10	-
To feel more self confident	5	6	-
To motivate themselves for therapy	1	10	-
As a strategy training	4	6	1
Other reason	-	-	-
Do therapists think patients are able to imagine movements	-	11	-
			

	Yes	Don't know	No
Do therapists think patients benefit from mental practice (is MP beneficial to recovery)?	2	9	-

Should MP be part of the rehabilitation guidelines according to the therapists and will therapist apply MP after the trial has ended? MP part of guidelines?	1	9	1
Will apply MP after trial?	7	4	-

MP = mental practice

### Patients' experiences with the mental practice framework

Patients reported several ways that they were facilitated to learn and use imagery. Four participants reported through the questionnaire that observing the movement first in others was the most helpful way to learn to use imagery. Observing could then be followed by practicing together or breaking the movement up into small parts. Another four patients thought that 'talking through and verbalising the movement' was the most effective way to generate images. Other methods that assisted patients learning included: 'continuously repeating' and 'experiencing support from the therapists'. Three participants could not remember what helped most and one person did not answer this question (table 5).

**Table 5:** Perceived benefits and details on the use of mental practice reported by patients at the end of the six weeks intervention period

	Perceived benefits	Effort to perform imagery	Skills MP helped with	Skills MP did not help with	Intending to use it after six weeks	Facilitators for learning and using imagery
A-109	A lot	Easy	Arm movements, right foot | leg	N/A It always helped	Yes	When the therapists verbalises the movement during performance of the skill (not demonstrating or verbalising herself)
B-210	A lot	Easy	Walking stairs and getting up from the floor	N/A	Yes	Going through the steps before the skill performance, e.g. getting up from the floor
A-101	A lot	Neutral	Walking and becoming more secure	N/A	Yes	By experiencing the support and directions of the therapists
A-118	A lot	Neutral	Turning in bed, getting out of bed, walking	Washing and getting dressed	Yes	Demonstrations of the correct movement, observing others and chopping the movement up in small peaces
B-211	A lot	Neutral	Standing up from a chair	Keeping my balance	Not sure (not up to it yet)	Demonstrations and practicing together with the therapist
C-304	A lot	Hard	Walking	Did not use it for anything else but walking	No	I cannot remember which exercises or instructions facilitated use of MP most
C-306	A lot	Hard	Standing up from a chair and walking	N/A	No	I cannot remember which exercises or instructions facilitated use of MP most
A-114	Some	Easy	Walking with cane	N/A	Not sure	N/A
A-105	Some	Neutral	Walking, talking and putting on t-shirt	Talking; making S-sound	Yes	When the therapist explains the separate performance steps, lets me repeat them a lout and then lets me image the movement in my mind.
A-110	Some	Neutral	Standing up from a chair, walking with aids	N/A No idea	Yes	By chopping up the movement in parts and then repeat them (accompanied by cues/words, like 'left' and 'right')
B-206	Some	Neutral	Concentrating	N/A	Not sure	Demonstrations and practicing together with the therapists
B-207	Some	Hard	Standing up from a chair	With walking, but also because of bad knees	Not sure	Demonstrations
B-106	Some	Hard	Driving with wheel chair to the table	Transfer wheel chair to toilet	Yes	By continuously repeating
C-302	Some	Hard	Supination and pronation of the arm	N/A Did not use it for any other movements	No	I cannot remember which exercises or instruction facilitated use of MP most

MP = mental practice | N/A = not applicable | A-nnn = nursing home (A, B or C) and patient number

### Patients' opinion on short term perceived benefits and mental practice use

Table 5 shows the data collected from patients at the end of the six week intervention period concerning their use of and opinion on mental practice.

## Discussion

In the majority (eleven out of sixteen, 69%) of the participants in this study, the mental practice intervention was delivered according to the framework; patients received the minimum amount of mental practice recommended (thirteen out of fourteen, 93%), and they undertook unguided (twelve out of sixteen, 75%) practice as recommended.

Nonetheless, using the mental practice framework taught [11] in stroke patients in the sub-acute phase of recovery was more difficult than expected, and teaching and monitoring mental practice may need a longer and a more intensive training period than given in this study. The relatively long time needed to teach mental practice to patients may be due to reduced memory and concentration skills in our patients. In this context, it must be noted that Dutch nursing homes admit patients who are older, more frail and more cognitively impaired than those selected for specialist stroke rehabilitation centres. This may explain the lack of any effect seen in this study. Most other studies have been on younger and less frail patients. Whether the additional effort would be compensated for later as the patient continues unguided mental practice is uncertain because some patients do not practice outside of therapy at all and in these patients the advantage of teaching mental practice would most likely be lost [13].

When we developed the mental practice intervention we deliberately allowed therapists to tailor the intervention to the needs and abilities of the individual patient. This flexibility was considered important, to reflect normal clinical practice where therapy is tailored to each patient's situation, but this flexibility seemed to make it more difficult and confusing for the therapists. Although therapists were content about the training of the mental practice framework and knew what was expected, they would have liked to have had more certainties in order to know if they were delivering the protocol as described. The variation in the extent of use between therapists may also reflect difficulty in changing professional practice. Changing practice seemed more difficult for the physiotherapists than for the occupational therapists. On the other hand as shown in table 4, all therapists saw potential benefits from applying mental practice although half intended to use it after the trial, and some only intended to use parts of the framework.

The flexibility allowed in the mental practice training protocol also arguably made the research more difficult because there was less control over and knowledge about the very specific details of treatments given. This was added by the intrinsic difficulty in measuring different aspects of the mental practice intervention. Our approach has been that the randomised trial was investigating the effect of teaching a therapist to teach the patient about mental practice, and we therefore investigate (a) the process of teaching the patient and, as far as it is feasible (b) the direct effects of that teaching upon the patient's use of the technique. We have to acknowledge that we have not collected precise and certain data on every aspect; this was certainly not practical and is probably not possible.

### Methodological quality of the study - limitations

The small number of participating patients and relatively large number of therapists seeing these patients led to a large number of therapists being involved in treating relatively few patients so that the therapists had relatively few patients to gain familiarity with the techniques. This might have made therapists less confident, influencing their answers in the questionnaire. More importantly, they may not have learned how best to teach patients, again reducing the probability of having an effect.

Most other reported studies of mental practice have given (a) a fixed treatment and (b) minimal or no information on the content or perception of the treatment. There is only one other study reporting on the perception of patients of mental practice therapy [17], which reported that stroke patients were generally positive about applying mental practice. One other study [18] described the training of therapists. Some studies have checked compliance using a log [19,20], an interview [20], a phone call half way through [19] or self reported independent mental practice [18].

We could not find any published data on the opinions or experiences of therapists with a mental practice intervention. Two studies reported that patients were satisfied with the imagery intervention and that no problems with the rehearsals or generalisations occurred [18,20]. How this satisfaction exactly was established is not clear. If or how patients were instructed to use imagery is not or seldom described in published effect studies, with the exception of mental practice through audio taped cassettes [20].

No adverse effects of mental practice have been reported in stroke trials, and we did not observe any. Therapists sometimes stopped mental practice for a couple of days if they thought patients were 'thinking too much' and got irritated. However adverse effects have been reported in trials of mental practice as part of an intervention to reduce chronic limb pain [21].

Process evaluation of new and researched complex interventions is important [22] and to the best of our knowledge, this is the first detailed process evaluation of a mental practice intervention in neurological rehabilitation. This study suggests that it is possible to teach some patients who have had a stroke within the previous two-10 weeks how to use mental practice, and they will use it. Therapists may need specific additional support in learning how to tailor the teaching protocol to the individual needs of each patient and they should be given an opportunity to develop their new skills before any formal evaluative research is undertaken.

## Conclusions

We found that the explanation and teaching of mental practice to patients occurred in all patients, some use occurred in most patients but self-directed use was relatively less common. Therapists found the flexibility in the protocol difficult to manage, probably due to very limited exposure to and experience of the technique. Patients varied, with some finding it an easy and useful technique but others being less enthusiastic.

## Competing interests

The authors declare that they have no competing interests.

## Authors' contributions

All authors made substantial contributions to conception and design of the trial and process evaluation. SB and JVH developed the questionnaires. All authors read and approved the final manuscript.

## Pre-publication history

The pre-publication history for this paper can be accessed here:

http://www.biomedcentral.com/1471-2377/10/74/prepub
